# Development of a measure to assess the quality of proxy decisions about research participation on behalf of adults lacking capacity to consent: the Combined Scale for Proxy Informed Consent Decisions (CONCORD scale)

**DOI:** 10.1186/s13063-022-06787-8

**Published:** 2022-10-04

**Authors:** Victoria Shepherd, Kerenza Hood, Katie Gillies, Fiona Wood

**Affiliations:** 1 Centre for Trials Research, Cardiff University, Cardiff, UK; 2 Health Services Research Unit, University of Aberdeen, Aberdeen, UK; 3 PRIME Centre Wales, Cardiff, UK; 4 Division of Population Medicine, Cardiff University, Cardiff, UK

**Keywords:** Informed consent, Clinical trial, Proxy, Decision-making, Measure, Comprehension

## Abstract

**Background:**

Recruitment of adults lacking the capacity to consent to trials requires the involvement of an alternative ‘proxy’ decision-maker, usually a family member. This can be challenging for family members, with some experiencing emotional and decisional burdens. Interventions to support proxy consent decisions in non-emergency settings are being developed. However, the ability to evaluate interventions is limited due to a lack of measures that capture outcomes of known importance, as identified through a core outcome set (COS).

**Methods:**

Using established measure development principles, a four-stage process was used to develop and refine items for a new measure of proxy decision quality: (1) findings from a recent scoping review and consensus study were reviewed to identify items for inclusion in the scale and any existing outcome measures, (2) assessment of content coverage by existing measures and identification of insufficiency, (3) construction of a novel scale, and (4) cognitive testing to explore comprehension of the scale and test its content adequacy through interviews with family members of people with impaired capacity.

**Results:**

A range of outcome measures associated with healthcare decision-making and informed consent decisions, such as the Decisional Conflict Scale, were identified in the scoping review. These measures were mapped against the key constructs identified in the COS to assess content coverage. Insufficient coverage of areas such as proxy-specific satisfaction and knowledge sufficiency by existing instruments indicated that a novel measure was needed. An initial version of a combined measure (the CONCORD scale) was drafted and tested during cognitive interviews with eleven family members. The interviews established comprehension, acceptability, feasibility, and content adequacy of the scale. Participants suggested re-phrasing and re-ordering some questions, leading to the creation of a revised version.

**Conclusions:**

The CONCORD scale provides a brief measure to evaluate the quality of decisions made on behalf of an adult who lacks the capacity to consent in non-emergency settings, enabling the evaluation of interventions to improve proxy decision quality. Initial evaluation indicates it has content adequacy and is feasible to use. Further statistical validation work is being undertaken.

**Supplementary Information:**

The online version contains supplementary material available at 10.1186/s13063-022-06787-8.

## Background

Participants included in clinical trials often do not reflect the entire population that the intervention is intended to be used for, leading to concerns about both the generalisability of the results and the impact on excluded groups who are under-served by research [1]. One such under-served group is adults who have an impaired capacity to consent to research due to, for example, a neurodegenerative condition such as dementia, an acute or critical illness, or a life-long disability [1]. The exclusion of adults who lack the capacity to consent from trials in non-emergency settings is widespread, even in populations with a high prevalence of cognitive impairment, and so inclusion might be particularly expected [2]. This has been found in trials in populations ranging from people with hip fractures [3, 4], older people [5], and people with intellectual disabilities [6].

Whilst the barriers to the inclusion of adults lacking capacity are complex and multifactorial, one of the principal factors is that conducting research with people with impaired capacity to consent relies on alternative decision-makers to provide informed consent on their behalf [7]. Where someone lacks the capacity to consent, family members are usually approached to act as a ‘proxy’ or ‘surrogate’ decision-maker [8, 9]. However, it can be difficult for families to make a decision about whether their relative should participate in a research study, and many experience an emotional and decisional burden as a result [10]. Families often express uncertainty about making what for some can be complex and challenging decisions, which can lead to psychological stress when asked to take on this role [11, 12]. Proxy decision-making for research has been demonstrated to be particularly stressful in some settings and contexts [13], with some studies reporting that nearly all proxies experience some degree of burden when making decisions about research [14]. This contributes to a higher proportion of families declining participation than patients themselves [15]. Despite numerous innovations to improve informed consent processes for research, there are currently no effective interventions for proxies who are making decisions on behalf of someone who lacks capacity in either emergency or non-emergency situations, although these are currently under development.

### Evaluating interventions to support proxy consent

One intervention recently developed to support proxies in making decisions about non-emergency research participation is a decision aid (DA) intended to help families to make more informed and supported decisions when acting as a proxy [16]. Using decision science principles, DAs can help when making complex and preference-sensitive decisions, including decisions about participating in clinical trials [17, 18]. DAs differ from traditional information materials in that they do not focus solely on improving the delivery of information [18], but instead are intended to facilitate decision-making and lead to decisions which are more informed and consistent with the person’s values [19]. We developed a DA for proxy decisions about trial participation in response to family members identifying a need for better information and support when making these challenging decisions [16]. The DA has undergone acceptability testing with family members of people with impaired capacity to consent and now requires evaluation to determine if it does provide an effective form of support. Establishing the effectiveness of DAs (compared to standard approaches or alternatives) requires evidence that they improve decision quality—that is the dual constructs of both the quality of the decision-making process and the quality of the choice made [20]. There was therefore a need to develop an appropriate outcome measure to assess the quality of proxy decisions made about research participation in order to evaluate the DA and similar future interventions [16].

### Developing an appropriate outcome measure

The first step in the development process of any new scale is to establish a theoretical definition of the focal concept [21]. A concept synthesis approach was used to undertake the first conceptualisation of the construct - what constitutes a high-quality proxy consent decision [22]. Following this, a scoping review and consensus study (COnSiDER Study) was conducted to establish the core outcomes that are important to stakeholders when evaluating interventions to improve proxy decisions about research [23]. The final core outcome set (COS), which was developed with an expert stakeholder Delphi panel (patients/public and those who care for them), consists of 28 items across 11 domains including knowledge sufficiency, values clarity, self-efficacy, preparedness, and satisfaction [23]. Once a COS has been agreed, the next stage in COS development is to determine how the outcomes that are known to be important should be measured [24]. This paper builds on the COS to report the development of the Combined Scale for Proxy Informed Consent Decisions (CONCORD scale).

## Methods

**Fig. 1:**
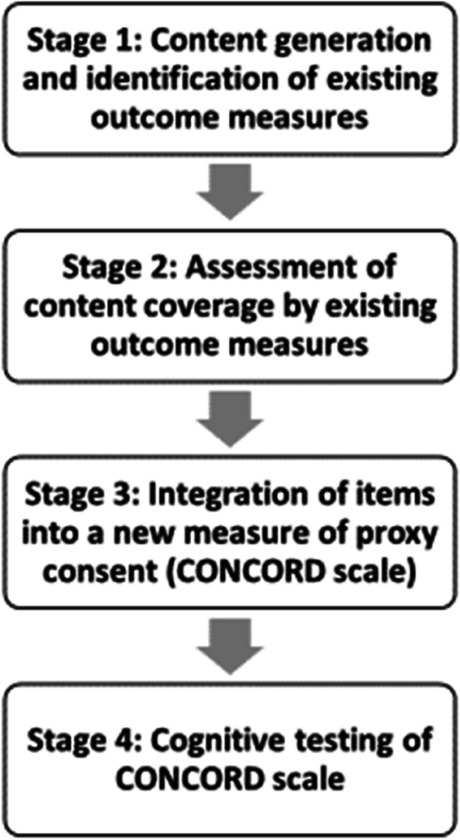
CONCORD scale development process

The process of determining how the outcomes included in the COnSiDER COS should be measured was conducted in four sequential stages based on established measure development principles [25]. The development process is shown in Fig. 1. When devising items for a new tool, the development stages are as follows: (1) identification of the content to be included, (2) review of previous work to determine if existing outcome measures are adequate and comprehensively cover the construct domains being measured, (3) this is then followed by the development of new items, and (4) assessment such as face validity and comprehension of the new measure and the feasibility of using it [25].

### Stage 1: content generation and identification of existing outcome measurement instruments

During the scoping review which was conducted as part of the COS development (published previously [23] including details of the methods and results), studies reporting the evaluation of decision support interventions to either improve consent in trials or proxy decision-making for care/medical treatment were reviewed. Data related to the outcome domains assessed were extracted to inform ‘what’ should be measured by the COS. Data relating to any outcome measurement instruments (OMI) used were also extracted in order to establish ‘how’ the outcome should be measured [26]. As there were no OMI specific to the assessment of proxy consent decisions, the closest were for decision aids intended for patients making decisions about healthcare (and more recently trial participation) and decision aids intended for proxies making non-research decisions, such as decisions about place of care on behalf of someone living with dementia.

### Stage 2: assessment of content coverage by existing outcome measurement instruments

The aim of the second stage was to identify candidate outcome measures and assess content coverage. This was done through the development of a matrix which can help to determine how representative items are across content domains for a concept under measure [25]. Within the matrix, items identified in the COS were mapped against the validated scales used in the studies included in the scoping review and in the wider DA literature such as a recent update of a Cochrane review of DAs for people making treatment or screening decisions [27]. These constructs were then tabulated against those included in relevant validated OMI including the Decisional Conflict Scale (DCS) [28], Quality of Informed Consent Scale (QuIC) [29], Satisfaction with Decision Scale (SWDS) [30], and the Decision Regret Scale (DRS) [31]. Any overlapping areas, or where constructs were not sufficiently covered by these existing measures, were identified.

### Stage 3: construction of a novel combined measure of proxy consent

The lack of coverage by, and relevance of, existing OMI to this novel construct (quality proxy consent decisions) meant that a new scale was needed. This could take the form of an additional scale to supplement the existing OMI, or a new combined scale that could be used as a single OMI. Integrating scales can be problematic, for example where they differ in terms of the response format [32]. However, the benefits of having one combined measure in terms of the ability to establish a consistent and reliable measure of specific relevance to this construct, and to reduce the burden of completion when administered to family members acting as proxy during a difficult time, led to the decision to develop a single combined multi-dimensional OMI—the Combined Scale for Proxy Informed Consent Decisions (CONCORD scale).

The construction of the combined measure followed the guiding principles and methods used in Steiner et al. who describe the derivation of items from other widely used indices, with modifications where needed, and the addition of items to meet the requirements of the new scale [25]. This enables the use of previously tested and psychometrically sound items, although their terminology may require updating, alongside the inclusion of new items which may come from sources such as research involving the ‘target population’ themselves [25].

A draft version of the CONCORD scale was developed, in preparation for piloting in the stage 4 of the project. The items were drafted by the first author with reference to the corresponding core outcome set item and then reviewed by the research team and iteratively revised. All items should be worded simply and unambiguously [33]; therefore, the readability and comprehension of the questionnaire was checked using a software tool (QUAID—question-understanding aid) to identify and improve issues around the wording, syntax, and semantics of questions [34]. This included revising any double-barrelled questions. Previous research suggests that Likert-type response scales with 5 to 7 points generally have better reliability and sensitivity than responses with 2 to 3 points [35]. Therefore, CONCORD consists of Likert-type scales with 5 points on the response scale, across its 28 items.

### Stage 4: cognitive testing of the CONCORD scale

Best practice guidance for developing and validating scales suggests that a draft questionnaire should be administered to 5–15 interviewees in 2–3 rounds whilst allowing participants to verbalise the mental process entailed in providing answers [33]. Therefore, the final stage of this project was to cognitively test and refine the CONCORD scale with family members of people living with an impairing condition such as dementia, prior to its use in a future evaluation of a decision support intervention [36]. The aim of the cognitive interviews was to obtain a range of views and experiences of family members of someone with a condition that affects (or may affect) their ability to provide consent to participate in non-emergency research. This included a variety of conditions and relationships (e.g. spouse, child/parent).

Cognitive testing is concerned with how people interpret and comprehend questions, recall information and events, make judgements about how to respond, and provide a response [37]. When developing measurement instruments, it can enable an understanding about whether participants can understand the question concept, whether they do so in a consistent way, and in a way the researcher intended [38]. Cognitive interview methods include the use of ‘probing’ to elicit how the participant went about answering the question and to explore how easy or hard the participant found it to answer the question [38]. Participants were asked to imagine themselves in a hypothetical scenario where they were being asked to act as proxy on behalf of their family member with dementia. They were asked to complete the questionnaire taking as long as they thought they would need, followed by semi-structured questions to explore their responses and understanding of each item.

#### Participant recruitment

Participants were identified via social media platforms such as Twitter and through Join Dementia Research (JDR). JDR is an online registry that enables volunteers with memory problems or dementia, carers of those with memory problems or dementia, and healthy volunteers to sign up and register their interest in taking part in research [39]. Participants who expressed an interest in participating were provided with a Participant Information Sheet and consent form by post, together with a copy of the CONCORD scale contained in a separate sealed envelope.

Cognitive interviews were conducted via an online video conferencing platform (Zoom) which has been shown to be a reliable tool for collecting qualitative data [40]. Verbal consent was obtained prior to the start of the interview, and before the study commenced it received a favourable ethical opinion from the School of Medicine Research Ethics Committee at Cardiff University. The target sample size was informed by the literature reporting the development of health measurement scales [25]. Interviews were conducted over 2 rounds. In round 1, cognitive interviews explored the initial draft scale, with a revised version of the scale being developed following an interim analysis of the interviews. Round 2 explored the revised version with a different cohort of participants.

#### Data collection

Interviews comprised of two parts. Firstly, the participant was provided with a brief scenario—to imagine that the person they cared for was being considered for a research study (emphasising that it could be any type of study such as a drug trial or a music therapy intervention) and they were being approached to help make a decision about whether they should participate or not and were then provided with the questionnaire. They were then asked to open the sealed envelope containing the CONCORD scale and asked to complete it as if they had just acted as a consultee or legal representative. The time taken for completion was recorded by the researcher. Secondly, immediately after completing the scale the participant was asked about their understanding of the individual questions and their views about the scale as a whole. A topic guide containing questions about the participant’s views and experience of completing the scale with standardised ‘probes’ or prompts was used as the basis for the interviews. Particular attention was paid to any items that appeared to elicit greater uncertainty or misunderstanding. Interviews were conducted by the lead researcher (VS) and audio-recorded with consent and transcribed verbatim prior to analysis. The transcripts were checked for accuracy and completeness against the source data.

#### Data analysis

Interview transcripts were read and re-read to ensure familiarity and inductively coded. Inductive analysis is a data-driven approach which can enable a rich description of the overall data to be provided [41] and has been used in previous studies using cognitive interviews [42]. NVivo software (v.12) was used to organise and store the data and support data analysis. The coding framework was reviewed by the research team, and consensus on coding reached through discussion during the coding process. Reflexivity is key to qualitative research [43] and developments in the analytical process were recorded through data analysis memos held in NVivo. Interviews were conducted until it was considered that data saturation had been reached, defined as adequacy of the data generated (in terms of richness and complexity) and is the point at which no new information, codes, or themes are yielded from the data [44], as determined through agreement between the study team. The CONCORD scale was then revised and finalised in preparation for use in a study to evaluate a decision aid for proxies, with concurrent validation of the scale [36].

## Results

### Stage 1: content generation and identification of existing outcome measurement instruments

Searches of published literature identified 14 studies that met the criteria for inclusion in the scoping review. The full results of the searches can be found in the published scoping review [23]. Characteristics of the studies and the decision-related outcome measurement instruments used in the studies are shown in Table 1.

**Table 1:** Characteristics of studies included in the scoping review and decision-related outcome measurement instruments used

Lead author name	Publication date	Setting	Type of decision	Self or proxy decision	Outcome domains	Outcome measurement instruments^**a**^
Juraskova, I et al. [36]	2015	Oncology Australia and New Zealand	Participation in breast cancer trial	Self	Anxiety/depression; attitudes towards participating; decisional conflict; involvement preferences; actual (objective) understanding; perceived (subjective) understanding	Including: adapted information style questionnaire; CPS; DCS; QuIC
Politi, MC et al. [37]	2016	Oncology USA	Participation in cancer trial (multiple cancers and trials)	Self	Clarity of opinion about participating; decision readiness; decisional conflict; intent to participate; knowledge; self-efficacy	Including: questionnaire of eleven objective knowledge items; low literacy version DCS; decision readiness measured on a single-item 5-point scale
Sundaresan, P et al. [38]	2017	Oncology Australia and New Zealand	Participation in prostate cancer trial	Self	Anxiety/depression; decisional regret; decisional conflict; knowledge	Including: objective knowledge measured using adapted 11-point and 7-point knowledge scales; DCS; QuIC; MDMIC with additional items related to clinical trials; DRS; SWDS
Robertson, EG et al.	2019	Oncology Australia	Participation in acute lymphoblastic leukaemia trial (children and young people)	Self	Acceptability of DA; decisional conflict; emotional safety; feasibility; involvement in decision-making; knowledge	Including: preferred and actual decision‐making role (purpose designed); DCS for parents (purpose designed for adolescents), FCC‐HL‐AYA (adapted for parents and adolescents); adapted versions QuIC
Cox, C et al. [26]	2012	Intensive care USA	Prolonged mechanical ventilation provision in critical illness	Proxy	Acceptability of DA; conflict with physicians; decisional conflict; feasibility; physician-surrogate discordance; quality of communication; trust in physician; comprehension of relevant information	Including: QOC; DCS
Einterz, S et al. [27]	2014	Nursing homes USA	Treatment decisions for a person with advanced dementia	Proxy	Clinician–surrogate concordance; involvement in decision-making; knowledge; quality of communication; satisfaction with care	Including: QOC; knowledge assessed with 18 true/false items
Hanson, L et al. [28]	2011	Nursing homes USA	Feeding options in advanced dementia	Proxy	Clinician–surrogate concordance; decisional regret; frequency of communication with health care providers; involvement in decision-making; knowledge	Including: DCS; knowledge assessed using 19 true/false items; SWDS; DRS
Snyder, A et al. [29]	2013	Nursing homes USA	Feeding options in advanced dementia	Proxy	Decisional conflict; knowledge	Including: knowledge assessed using 19 true/false items; DCS
White, D et al. [33]	2012	Intensive care USA	Decisions about treatment options in critical illness	Proxy	Acceptability of DA; decisional confidence; feasibility; perceived effectiveness of DA; quality of communication; self-efficacy	Including: QOC; DSES; DCS
Cox, C et al. [34]	2019	Intensive care USA	Decision about prolonged mechanical ventilation provision in critical illness	Proxy	Anxiety/depression; clinician–surrogate concordance; decisional conflict; perception of care centeredness; quality of communication; comprehension of relevant information	Including: QOC; DCS
Hanson, L et al. [35]	2017	Nursing homes USA	Treatment decisions for a person with advanced dementia	Proxy	Advance Care Planning problem score; satisfaction with decision; decisional conflict; involvement in decision-making; quality of communication; satisfaction with care	Including: QOC
Lord, K et al. [30]	2017	Memory clinics UK	Dementia family carers deciding about place of care	Proxy	Acceptability of DA; anxiety/depression; decisional conflict	Including: DCS
Malloy-Weir, LJ et al. [31]	2017	Nursing homes Canada	Initiation of antipsychotic medications for a person with dementia	Proxy	Satisfaction with decision; decisional conflict; knowledge	Including: knowledge assessed using an 8–10-item survey based on the Ottawa Knowledge Questionnaire; low-literacy DCS; SWDS
Mitchell, SL et al. [32]	2001	Acute care Canada	Placement of a percutaneous endoscopic gastrostomy tube for older adult > 65 with cognitive impairment	Proxy	Acceptability of DA; decisional conflict; knowledge	Including: knowledge assessed in a multiple-choice format; DCS

*CPS* Control Preferences Scale, *DCS* Decisional Conflict Scale, *QuIC* Quality of Informed Consent Scale, *MDMIC* Multi-Dimensional Measure of Informed Choice, *DRS* Decisional Regret Scale, *SWDS* Satisfaction with Decision Scale, *FCC‐HL‐AYA* Functional, Communicative, Critical Health Literacy scale, *QOC* Quality of Communication scale, *DSES* Decision Self-Efficacy Scale

^a^Restricted to outcome measurement instruments for decision-related outcomes only

There was notable heterogeneity in the outcomes and OMI used. All studies used a combination of purpose-designed measures, some of which had been used in previous studies, and established and validated OMI. Of validated measures of decision quality, including the quality of decision support, the Decisional Conflict Scale (DCS) was most commonly used (12 (86%) studies), followed by Quality of Informed Consent Scale (QuIC) [29] and Satisfaction with Decision Scale (SWDS) [30] which were both used in 3 (21%) studies. DCS use included the traditional version with 16 statements and 5 response categories [28], as well as the low literacy version with 10 statements and 3 response categories [45]. The Decision Regret Scale (DRS) [31] was used in 2 studies. There was considerable heterogeneity in measures used to assess constructs such as objective and subjective knowledge, with most studies using purpose-designed scales containing decision-specific knowledge items.

### Stage 2: assessment of content coverage by existing outcome measurement instruments

The COS consists of 28 outcome items which relate to the process of decision-making, proxies’ experience of decision-making, and factors that influence decision-making such as understanding [23]. These cover key construct domains such as values clarity (understanding the personal value of options), subjective understanding (feeling informed), objective understanding (being informed), preparedness to make a decision, and regret and satisfaction with the decision—which included outcomes of that decision.

These constructs were tabulated against those included in commonly used validated measures including the DCS, QuIC, SWDS, and DRS. A focused literature review of outcome measures used in the evaluation of interventions to improve decision-making about healthcare and informed consent decisions identified additional candidate OMIs: DelibeRATE for measuring deliberation during the informed consent process for clinical trials [46], Preparation for Decision Making (PrepDM) scale [47], and Decision Self-Efficacy scale (DSE) [48]. As these were validated measures that are widely used in studies to evaluate decision aids, and form part of the well-established evaluation toolkit for the Ottawa Decision Support Framework (ODSF, a framework that aims to conceptualise the support needed for making difficult preference-sensitive decisions), the quality of the OMI was not formally assessed as is usually recommended when selecting outcome measurement instruments for outcomes included in a COS [26]. Any overlapping areas or constructs, and those not currently captured by these existing measures, were identified.

Across 28 outcome items, measures were identified for 18 of the outcomes when assessed using seven existing OMI. There was sufficient coverage of domains such as self-efficacy with all three items measured by existing scales. However, ten of the COS items across five domains (knowledge, understanding, deliberation, values congruence, and satisfaction) were not covered by any of the measures. This, unsurprisingly, included proxy decision-making-specific domains such as knowledge sufficiency—both about their role as proxy decision-maker and in relation to them knowing about the wishes and preferences of the person they are representing. Another domain not well covered was satisfaction, including whether the proxy felt that they had sufficient time to make a decision which is considered an essential component of informed consent for research [49]. There was considerably heterogeneity in the phrasing of many of the items, which primarily reflected the diverse origins of the scales included in this review.

### Stage 3: integration of items into a new measure of proxy consent

Based on the content gaps across existing items and the lack of specificity and applicability to proxy consent decisions described in stage 2, new self-report items were generated for the ten outcome items not already covered. Having identified the need for the newly developed items to be combined into a new scale alongside those adapted from existing OMI, it was recognised that a degree of harmonisation in the phrasing of individual items was needed in order to present a single combined questionnaire and improve comprehension. The phrasing of items which were covered by existing OMIs was slightly modified from the original COS wording to more closely align with one another and to improve comprehension when used as a self-completed OMI rather than as a list of COS items. A draft version of the CONCORD scale was developed.

In order to establish initial face validity prior to larger scale testing, this draft version of the scale then underwent initial testing with a small group of lay advisors (*n* = 3) who support the larger research programme. This resulted in changes being made to the layout of the questionnaire so that it was easier to navigate, including grouping items into three sections with headings to help orientate respondents towards which stage of the decision-making process they were being asked about. Three sections were created: preparation for decision-making, decision-making process, and decision outcome. By the end of this stage, the first test version of the CONCORD scale (version 1.0) was developed.

### Stage 4: cognitive testing of the CONCORD scale

Remote cognitive interviews were conducted with eleven family members of someone living with dementia between September and October 2021 (round 1) and between November and December (round 2) with the revised version of the scale (version 2.0). The mean duration of interviews was 43 min (range 29–59 min). Participant characteristics are presented in Table 2.

**Table 2:** Cognitive interview participant characteristics

	Round 1 participants *n* = 6 (%)	Round 2 participants *n* = 5 (%)	Total participants *n* = 11 (%)
Sex^a^			
Male	2 (33%)	1 (20%)	3 (27%)
Female	4 (67%)	4 (80%)	8 (73%)
Age			
20–29	0	1 (20%)	1 (9%)
30–39	2 (33%)	0	2 (18%)
40–49	0	1 (20%)	1 (9%)
50–59	2 (33%)	1 (20%)	3 (27%)
60–69	1 (17%)	2 (40%)	3 (27%)
70+	1 (17%)	0	1 (9%)
Country			
England	2 (33%)	5 (100%)	7 (64%)
Wales	4 (67%)	0	4 (36%)
Ethnicity			
British (White)	4 (67%)	5 (100%)	11 (100%)
Not stated	2 (33%)	0	0

^a^Sex registered at birth. All participants described their gender as being the same as the sex they were registered at birth

An interim analysis was conducted after round 1, and the results were discussed among the research team. Where responses indicated uncertainty or participants had recommended specific word changes to items to increase clarity, a consensus was reached about which modifications to the content or additions were required. This included changes to the phrasing of some questions to reduce uncertainty about what decision was being referred to, through adding the term ‘decision about my relative taking part in the study’, and to ensure greater consistency instead of using a combination of ‘choice’, ‘option’, and ‘decision’. Changes were also made to the order of questions in the section concerning preparation for decision-making, where some round 1 participants felt they had lacked a ‘logical’ order. Revisions were made to the wording and ordering of the CONCORD scale as a result. The revised version was then used in the interviews in round 2. Participants’ general views about the length and format of the questionnaire are reported in the following text, with corresponding illustrative quotes in a supplementary file (Additional file 1).

#### Instructions for completion of the CONCORD scale

Participants appeared to understand the brief instructions for completing the scale that were provided at the beginning of the questionnaire, with no participants requiring further explanation to complete the questionnaire. However, when specifically asked about the clarity of the instructions, many participants had not read them in detail, appearing to skip over them to complete the questions.

#### Length and format of the questionnaire

The mean time for completion of the questionnaire was just under 3 ½ min (range 1 ½ to 5 min). All participants considered the questionnaire to be of reasonable length. One participant commented that proxy decision-makers are also likely to be (or have been) carers and so will be experienced in completing forms as part of that carer role, including much longer administrative questionnaires. The format was considered to be acceptable, although one participant commented that the font size may be a little too small, for example for people with visual impairments.

#### Ordering of items

Following the changes made to the order of the items after round 1 interview, participants in round 2 considered the order of the three sections and the items within each part to be acceptable and have a ‘logical order’ that followed their thought processes. Suggestions were made to further revise the section headings to more clearly describe the content of each section and to consider labelling them as A–C, although, as with the instructions at the start of the questionnaire, not all participants read the section headings closely or were conscious of having done so.

#### Views about the contents and acceptability of the questionnaire

Participants understood the purpose of the questionnaire, and all viewed the contents of the questions as being acceptable or viewed as ‘pretty harmless’. Whilst participants generally felt that items were clear and straightforward, their responses in round 1 indicated that some items included terms that required further explanation, e.g. what form ‘support’ might take when asked if they had enough support to make a decision.

Other responses indicated that quantifying sufficiency was challenging—particularly against the backdrop of uncertainty that surrounds proxy decision-making. For example, some participants questioned whether they could ever feel confident or satisfied with their decisions. Some participants identified points of redundancy across a small number of the items, for example ‘I feel it is the right decision’ and ‘I feel the decision was a wise one’ where participants felt that ‘right’ and ‘wise’ were similar concepts. However, other participants viewed them as disparate items and considered their responses to whether their decision was right or wise to be distinct from one another.

Participants often distinguished between questions that related to their own feelings and knowledge, and those that required thinking about the wishes of the person they were representing—which they described as requiring more time and consideration. Some participants described the impact that completing the questionnaire had on their (hypothetical) decision-making and how it prompted them to think about some of the issues. When asked about areas they thought were important but missing from the questionnaire, no participants identified any areas of content inadequacy.

#### Scoring

All participants attempted to complete the CONCORD scale, although it was a counterfactual exercise for participants and so some questions were slightly more difficult for some. One participant [ID 06] did not complete a small number of items (*n* = 3) where the meaning was not clear to them, and another [ID 01] did not complete a larger number of items (*n* = 14) as they focused on commenting about the phrasing and order of items which they felt meant that they could not score those items. Some participants said that they had scored an item as ‘neither agree nor disagree’ where they were not sure of the meaning, although more normally they used this to indicate a neutral response.

#### Creation of a final version

Following the completion of round 2, the results were discussed among the research team. Additional minor revisions were made to the wording of a small number of items where participants appeared to be uncertain about the meaning or where they suggested it might be unclear for others. The refined version of the CONCORD scale is shown in Table 3, with a 5-point response scale where 1 = strongly agree to 5 = strongly disagree. Responses are then reverse scored, with higher scores indicating higher decision quality.

**Table 3:** Combined Scale for Proxy Informed Consent Decisions (CONCORD scale)

**Part A. Thinking back to when you made the decision, how informed did you feel?**	
1	I am informed about the purpose of the study, any procedures, risks and benefits
2	I have been informed about my role in making the decision about my relative taking part in the study
3	I am able to represent my relative’s wishes and preferences
4	I am clear about which benefits from taking part (for them or others) would matter most to my relative
5	I am clear about which disadvantages of taking part would matter most to my relative
6	I am clear whether the benefits or disadvantages of taking part would be more important to my relative
**Part B. How did you feel during the process of making a decision?**	
7	I recognise that a decision about my relative taking part in the study needs to be made
8	I understand the information that I need in order to make a decision
9	I understand that the decision about taking part in the study depends on what would matter most to my relative
10	I feel that I am adequately informed about the issues that are important to my relative
11	I feel able to ask the research team any questions I have about the study
12	I feel able to express my opinions about my relative taking part in the study or not
13	I feel as involved as I want to be in the decision
14	I feel that the information about the study prepared me to make a decision
15	I feel confident that I can understand the information well enough to make a decision
16	I have given the decision about whether my relative takes part or not consideration and thought
17	I am clear about what my relative’s wishes and preferences would be about taking part in the study
18	I feel supported to make a decision about the study
19	I am confident that I can make a decision about the study
20	I feel confident that I can delay my decision if I need more time
21	I am ready to make a decision about the study
**Part C. How do you feel about the decision you have made?**	
22	I am satisfied with my decision
23	I am satisfied that my decision would be consistent with my relative’s values
24	I feel it is the right decision
25	I feel the decision was a wise one
26	I feel I had enough time to make a decision
27	I am comfortable with the decision
28	I feel that the decision process was good (regardless of the outcome)

## Discussion

Building on previous conceptual and empirical work, this paper reports on the development and first phase of the validation for the CONCORD scale, a self-reported combined measure of the quality of proxy consent decisions made in non-emergency settings on behalf of other adults. Higher scores indicate a higher quality decision, defined as one in which the proxy is prepared and supported to make a decision and feels satisfied they have made a decision based on the preferences of the person they are representing, and where they accept the uncertainty they may experience [22]. This novel questionnaire is intended to measure a complex and—when compared to decisions about healthcare or participation in a trial made for oneself—relatively under-developed construct. This makes comparisons with existing scales difficult. However, the length of the questionnaire and time for completion is broadly similar to other scales measuring aspects of informed consent for clinical trials. This includes the Quality of Informed Consent (QuIC) questionnaire which measures participants’ understanding of cancer clinical trials using 34 questions with three response levels and requires an average of 7.2 min to complete [29]. The DelibeRATE questionnaire measures deliberation during the informed consent process for clinical trials and consists of ten items to be rated on a three-level Likert scale and takes approximately 5 min to complete [46]. It is also broadly comparable with OMI used in studies evaluating proxy decisions that were included in the scoping review (see Table 1). These typically used a combination of scales such as Decisional Conflict Scale (DCS) [28] which consists of 16 items plus an option preference question, alongside a knowledge assessment using a study-specific questionnaire with approximately 19 items.

The scale underwent cognitive testing which demonstrated that participants had strong comprehension of the items. Whilst the current version of the scale consists of 28 items, the time required for completion by participants was low, and the high levels of acceptability reported suggest feasibility. Although no areas of content inadequacy or redundancy were identified, the scale has yet to undergo formal statistical evaluation, following which items may be removed due to redundancy. Therefore, in line with the recommended practice for developing and validating scales, some content that is currently included may ultimately be shown to be either redundant or unrelated to the core construct [33]. Further work is also needed to inform the scoring instructions for CONCORD, including any weighting of the items and transforming the raw score to a 0–100 scale.

A future evaluation study is planned as part of the larger programme of research that this project forms part of. This will include establishing the feasibility of using the CONCORD scale in ‘real’ (rather than hypothetical) proxy consent decision-making contexts, with concurrent validation of the scale as part of a ‘Study Within a Trial’ (or ‘SWAT’) to evaluate the effectiveness of a decision aid for families making proxy consent decisions across a number of host trials [36]. Family members will be randomised to receive the decision aid alongside the standard information about the trial they would receive as consultee or legal representative, or the standard information alone [50]. This research programme has also included developing an empirical and conceptual account of proxy consent decisions made by families [51], development of the COS [23], and the decision aid intervention [16]; therefore, this further evaluation work will also contribute to the content validation of this scale and developing the theory that underpins this work.

Further research is also needed to explore CONCORD as a measure of the quality of other types of proxy decisions relating to research, such as participation in emergency research, the decision to withdraw a participant who lacks capacity, or decisions made on behalf of children.

### Strengths and limitations

A strength of our study is that the scale development process was based on established measure development principles. Other strengths include the involvement of an expert stakeholder panel in the COS which led to the generation of content items and contributed to establishing the content validity of the scale. Although a relatively modest number of interviews were conducted, this is in line with best practice guidance [33] and data saturation was reached [44]. Conducting an interim analysis during the cognitive interview study meant that the revised version could undergo testing prior to a final version being produced ready for further validation work.

There are also some limitations to note. First, whilst the development of the scale is underpinned by empirical research involving families who have acted as a consultee or legal representative, the feasibility and acceptability of the scale has only been established in the context of hypothetical decision-making. Framing effects and any potential differences between real and hypothetical decisions remain the subject of much debate [52]. Secondly, as the scale is intended for proxy decisions regarding non-emergency research, it is not considered applicable to research decisions made during life-threatening and time-critical situations which are likely to differ. Lastly, the scale draws on work conducted in England and Wales with predominantly white participants and so, whilst there can be confidence in its validity in the UK and broadly similar contexts, the legislative and cultural differences surrounding the ethics and practice of proxy consent must be taken into account when considering the use of CONCORD in other jurisdictions.

## Conclusion

CONCORD is a 28-item scale to assess the quality of decisions about non-emergency trial participation made by family members on behalf of someone who lacks the capacity to consent for themselves. This new brief measure has the potential to help evaluate interventions that improve the quality of decisions made by proxies on behalf of someone who lacks capacity and reduce the emotional and decisional burden they experience as a result. Following further validation work, the use of this scale in future studies will enable meta-analyses to synthesise the growing literature on how best to support family members making decisions about research. Having a standardised approach to developing and evaluating interventions to improve proxy consent decisions will contribute to the developing evidence base in this novel area of research and so help to address some of the barriers to the inclusion of this under-served group.

## Supplementary Information

**Additional file 1.** Illustrative quotes from participants in Phase 4 cognitive interviews.

## Data Availability

The dataset generated and used in this study is available through the submission of a data request to the Centre for Trials Research at https://www.cardiff.ac.uk/centre-for-trials-research/about-us/data-requests.

## Abbreviations

COS: Core outcome set
CONCORD: Combined Scale for Proxy Informed Consent Decisions
DA: Decision aid
DCS: Decisional Conflict Scale
DRS: Decision Regret Scale
DSE: Decision Self-Efficacy scale
JDR: Join Dementia Research
ODSF: Ottawa Decision Support Framework
OMI: Outcome measurement instruments
PrepDM: Preparation for Decision Making
QuIC: Quality of Informed Consent Scale
SWAT: Study Within A Trial
SWDS: Satisfaction with Decision Scale (SWDS)
RCT: Randomised controlled trial
