# Supplementary material for: Development of a measure to assess the quality of proxy decisions about research participation on behalf of adults lacking capacity to consent: the Combined Scale for Proxy Informed Consent Decisions (CONCORD scale)
Source: Trials. 2022 Oct 4;23:843. doi: 10.1186/s13063-022-06787-8 (PMC9531498; doi:10.1186/s13063-022-06787-8)
Supplement: Supplementary file 1 — Additional file 1. Illustrative quotes from participants in Phase 4 cognitive interviews. [file 13063_2022_6787_MOESM1_ESM.docx]

***Additional file 1. Illustrative quotes from participants in Phase 4 cognitive interviews***

| **Views about the length of the questionnaire** |
| --- |
| *“I suppose I’m so used to just sort of doing this kind of thing that it was fairly rapid, because those questions which were about me and my decision, what I’ve got control over were very easy and quick and definite. But it’s the questions that are inviting me to pause and reflect on the relative’s point of view where people might take longer I guess.” [ID 01, Round 1]* |
| **Views about the ordering of items** |
| *“The question ‘I’m comfortable with the decision’ I think should come before ‘I feel that the decision process was good’…. simply because it leaves the questionnaire as a questionnaire, rather than how I feel - which is not so good.” [ID 06, Round 1]* |
| *“I think they were all logical. Some perhaps more searching than others but no, I think they were all in the right order because it takes you through the thought process.” [ID 08, Round 2]* |
| **Views about the contents and acceptability of the questionnaire** |
| *“The questions are pretty harmless. You know, it’s not asking you any personal information. It’s about how you found the process and how you feel about having to make this decision.” [ID 04, Round 1]* |
| *“But I think it’s good that those questions raise those issues, because it does prompt you to feel that you are weighing up the issues.” [ID 02, Round 1]* |
| *“I suppose that one perhaps feels similar to the last question. You know, in that if it's the right decision I suppose, in this circumstance, I'd hope that it would be also you know, wisely thought about.” [ID 10, Round 2]* |
| *“Different I think, I mean wise is broader I think somehow. …. one of them feels that it is more altruistic than the other and one is actually sensible, is it going to be asking too much or is it going to be too inconvenient and things like that. They all sort of unpick all of that a little bit.” [ID 07, Round 2]* |
| **Views about the scoring of items** |
| *“Now for that I’ve put I neither agree nor disagree because I wasn’t overly clear on that question.” [ID 08, Round 2]* |
